# Nontargeted Screening of Fingermark Residue Using Comprehensive Two-Dimensional Gas Chromatography–Time-of-Flight Mass Spectrometry for Future Use in Forensic Applications

**DOI:** 10.1021/jasms.5c00258

**Published:** 2025-09-09

**Authors:** Emma L. Macturk, Katelynn A. Perrault Uptmor

**Affiliations:** Nontargeted Separations Laboratory, Chemistry Department, 8604 William & Mary, Integrated Science Center 1053, 540 Landrum Drive, Williamsburg, Virginia 23188, United States

## Abstract

Fingerprints are routinely used as evidence in forensic investigations. Fingermarks, any mark left by a donor whether a complete print or not, include sweat and oil excreted by the donor. The chemical components of fingermarks are typically analyzed by gas chromatography–mass spectrometry (GC-MS). Complexity from the number of endogenous and exogenous components associated with fingermarks tends to cause challenging coelutions in resulting chromatograms. In these scenarios, nontargeted analysis can provide substantial benefits over traditional targeted methods that exist in the literature. In this proof-of-concept study, a nontargeted method for analyzing fingermarks was developed and optimized using comprehensive two-dimensional gas chromatography (GC×GC-TOFMS). Two different methods for extracting fingermarks off a microscope slide were evaluated for reproducibility and the quantity of extracted analytes, and a cotton swab collection with solvent extraction was chosen. Instrumental parameters were experimentally optimized to produce a final workflow. The optimized extraction and instrument methods together identified 70 fingermark analytes. Exogenous components within the deposited residue were resolved from endogenous fingermark compounds and used to differentiate donors based on personal care products used by the donor. The extra chromatographic space from GC×GC-TOFMS analysis was beneficial for resolving cosmetic compounds from endogenous fingermark compounds, some of which have been shown to coelute in GC-MS studies previously. The potential for a nontargeted screening of fingermarks for exogenous compounds in a forensic setting is demonstrated as an analysis of trace evidence.

## Graphical abstract

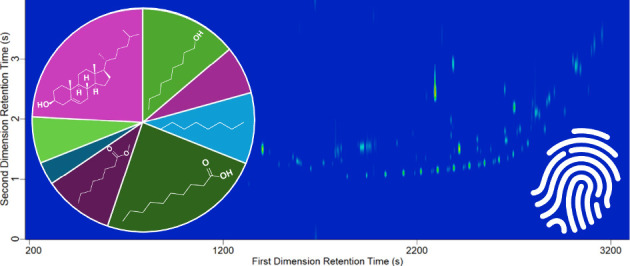

## Introduction

Deposited fingerprints are routinely used in forensic investigations to identify a suspect. Ridge characteristics and patterns of a deposited fingerprint are used to individualize the print to a suspected donor.^1^ Partial fingerprints that lack a complete ridge pattern often cannot be used for this individualization process.^2^ The fingerprint residue, or fingermark, contains traces of sweat and oil secreted by the donor’s sebaceous glands.^1,3^ The analyses of these organic components from the sebaceous glands have historically been conducted using gas chromatography–mass spectrometry (GC-MS) and inform how fingermark composition could advance suspect investigations in legal proceedings.^2–4^

Fingermark donor characteristics such as relative age and sex have been studied using various analytical techniques including GC-MS. Successful sex determination has been carried out using ultra high performance liquid chromatography–mass spectrometry (UPLC-MS), matrix-assisted laser desorption/ionization–mass spectrometry (MALDI-MS), and GC-MS to establish amino acid and lipid biomarkers.^2,5,6^ Relative donor age determination (i.e., whether a donor is a postpubescent adult or prepubescent child) has been studied with GC-MS using lipid ratios like cholesterol and squalene in fingermarks.^3,4^ This has been confirmed by two additional studies using Fourier transform infrared spectroscopy (FTIR) analysis which found more branched long chain fatty acids esterified with alcohols to be present in adults compared to children.^7,8^ Donor class identification studies of sex and relative age demonstrate forensic value in exploring the chemical profile of fingerprints. In addition to the compounds noted above that have been commonly identified in fingermark residue, fingermarks also have been identified as containing long chain fatty acids and fatty alcohols, wax esters, benzoic acid esters, and small vitamins.^9–13^

Contamination from exogenous compounds in fingermark residues based on the idea of touch chemistry could be used to associate an individual with a deposited fingermark. If an individual donor comes into contact with forensically relevant exogenous components, those compounds could be deposited in a fingermark left at a crime scene.^14,15^ Personal care products such as sunscreen and cosmetic products have been resolved from fingermark residue in the literature and their usage was found to be variable at the individual level.^5,16^ Sunscreen and insect repellent brands have been differentiated with MALDI-MS which would make lifestyle conclusions more specific to an individual who might buy one brand over another.^14^ Lifestyle choices including beauty products and dietary information have been shown to differentiate donors based on fingermark and other residue sampled from cellular phones.^17^

Although GC-MS systems have been used for these analyses, some challenges are noted in the literature. Certain cosmetic compounds have been shown to coelute with fingermark compounds such as cholesterol and vitamin E acetate.^9^ Also, in several studies, the high dynamic range and complexity of fingermark residue along with exogenous compound contamination is demonstrated within presented chromatograms, suggesting that the sample dimensionality may exceed the peak capacity of traditional GC-MS.^5,9,10,16^ Comprehensive two-dimensional gas chromatography with time-of-flight mass spectrometry detection (GC×GC-TOFMS) provides both an increased peak capacity and an increased analyte detectability. The technique uses a secondary column (^2^D column) of a different stationary phase than the first that increases compound resolution and would benefit the nontargeted analysis of fingermark residue. This would be especially useful in the case of authentic fingermarks that contain other external contaminants that individuals have encountered prior to depositing a fingermark.

GC×GC-TOFMS has been used to classify exogenous and endogenous compounds in human sweat, a matrix related to fingermark residue.^18^ GC×GC-TOFMS has also been used to differentiate individuals who have touched fired cartridges.^19^ Only one study has investigated clean latent fingermark residue using GC×GC-TOFMS.^20^ That study targeted seven fingermark analytes to compare analyses by 1D GC-MS and GC×GC-TOFMS. Although GC×GC-TOFMS was found to differentiate between fingermark sources better than 1D GC-MS, fingermark chromatograms of a clean, washed hand deposited prints with no coelutions by GC-MS analysis, and therefore no immediate benefit to GC×GC-TOFMS was found when looking solely at derivatized targeted components.^20^ However, the authors stated that GC×GC-TOFMS could be beneficial in forensic investigations for source attribution of exogenous compounds excreted from a suspect,^20^ though this has never been attempted experimentally in the literature. Additionally, a study which used GC-MS for fatty acid analysis from fingermarks also referred to the benefits of using GC×GC-TOFMS for this type of forensic application.^13^ A nontargeted method for resolving potential exogenous compounds, either excreted from the body or present through touch chemistry, could be a useful additional tool for forensic investigations of fingermark residue and seems to be desired by those conducting 1D GC-MS analyses in the literature.

In this study, a method for sample extraction and nontargeted analysis of the fingermark residue was developed and optimized using GC×GC-TOFMS. This method was then applied as a proof-of-concept study to the nontargeted screening of the fingermark residue for exogenous compounds (cosmetic products). The separation capacity and increased analyte detectability offered by GC×GC-TOFMS have the potential to provide substantial benefit in the nontargeted analysis of fingermark residue where endogenous fingermark components from sweat and oil can be resolved chromatographically from other exogenous components in the residue.

## Experimental Methods

### Subjects

All subjects gave informed consent for residue collection and sampling. Necessary steps were taken to protect volunteer identity and confidentiality. Sample collection and record keeping were conducted following Protocol PHSC-2024-03-05-16929-kaperrault approved by the William & Mary Protection of Human Subjects Committee. A single donor donated the residues used for method and sample extraction development. Four donors each donated three residues, and one donor donated six fingermark residues for the cosmetic application study.

### Sample Collection and Extraction

Subjects washed their hands for 20 s using unscented hand soap (Cole Palmer Essentials, Vernon Hills, IL) and then rinsed them for 5 s under deionized water. Hands were patted dry with paper towels. Subjects waited for 5 min without touching anything before they rubbed their middle three fingers (index, middle, and ring fingers) on their forehead for 10 s and deposited the residue onto a precleaned microscope slide (Avantor, Radnor, PA). For the initial extraction method, 200 μL of high-performance LC (HPLC)-grade dichloromethane (Thermo Scientific, Fair Lawn, NJ) was pipetted onto the surface of the microscope. The pipet tip was used to agitate the surface of the slide for 10 s, and then 100 μL of the aliquot was pipetted into a 2 mL GC vial (Restek Corporation, Bellefonte, PA) with a 300 μL insert (Thermo Scientific). Method blanks were prepared using the same extraction procedure on blank slides and then run under the same conditions to determine whether method interferences were present.

For the optimized extraction method, 200 μL of HPLC-grade dichloromethane (Thermo Scientific) was pipetted onto the surface of the microscope, and a sterile cotton tipped applicator was used to collect the residue and solvent off the slide (Dukal, Ronkonkoma, NY). The cotton swab tip was submerged in 500 μL of HPLC-grade dichloromethane (Thermo Scientific) in a 1.5 mL Flex Eppendorf tube (Eppendorf, Enfield, CT). The tube was vortexed for 10 s at 2000 rpm on a Thermo Scientific Basic Vortex Mixer. The swab was removed from the tube, a Spin-X basket (Corning Inc., Corning, NY) was added to the tube, and the swab was placed in the spin basket inside the tube. The Eppendorf tube with a spin basket insert was centrifuged for 5 min at 500 rpm on a Thermo Scientific mySPIN12. The sample was transferred to a 2 mL GC vial and evaporated using a MICROVAP Microplate Evaporator at 50 °C for 3 min at a 10 L/min nitrogen flow (Organomation, West Berlin, MA). The dried samples were reconstituted with 200 μL of dichloromethane and vortexed for 5 s at 2000 rpm and then transferred to a 2 mL GC vial with a 200 μL glass insert. All samples were injected for analysis within 1 h of collection to prevent sample loss or stored in a refrigerator at below 5 °C.

The following standards were acquired from Millipore Sigma: squalene (98%), cholesterol (98%), myristic acid (analytical standard), palmitic acid (pharmaceutical secondary standard), palmitoleic acid (98.5%), stearic acid (analytical standard), oleic acid (pharmaceutical secondary standard), methyl nonadecanoate (analytical standard), octisalate (analytical standard), and octocrylene (pharmaceutical secondary standard). d-α-Tocopheryl acetate (97%) was purchased from ThermoFisher. Standards were used for retention time matching when possible and for testing of recovery from sample preparation.

Three methods were tested for depositing fingermarks on the sample microscope slides. The three-finger method is described above, where subjects deposited an index, middle, and ring finger simultaneously for each hand. For single-finger depositions, subjects followed the same washing and drying procedure as above, then rubbed their middle three fingers on their forehead for 10 s before depositing the residue of each of their middle three fingers from both hands onto six individual precleaned microscope slides (i.e., 3 slides per hand). For replicates of the same finger, subjects washed their hands following the same washing, drying, and waiting procedure as directly above and then rubbed the middle fingers of both hands onto their forehead and deposited the residue onto a precleaned microscope slide. This process was repeated nine more times with the same two middle fingers from each hand for a total of ten replicate residues from the subjects’ middle fingers. The three deposition methods are visualized in Figure 1.

**Figure 1:**
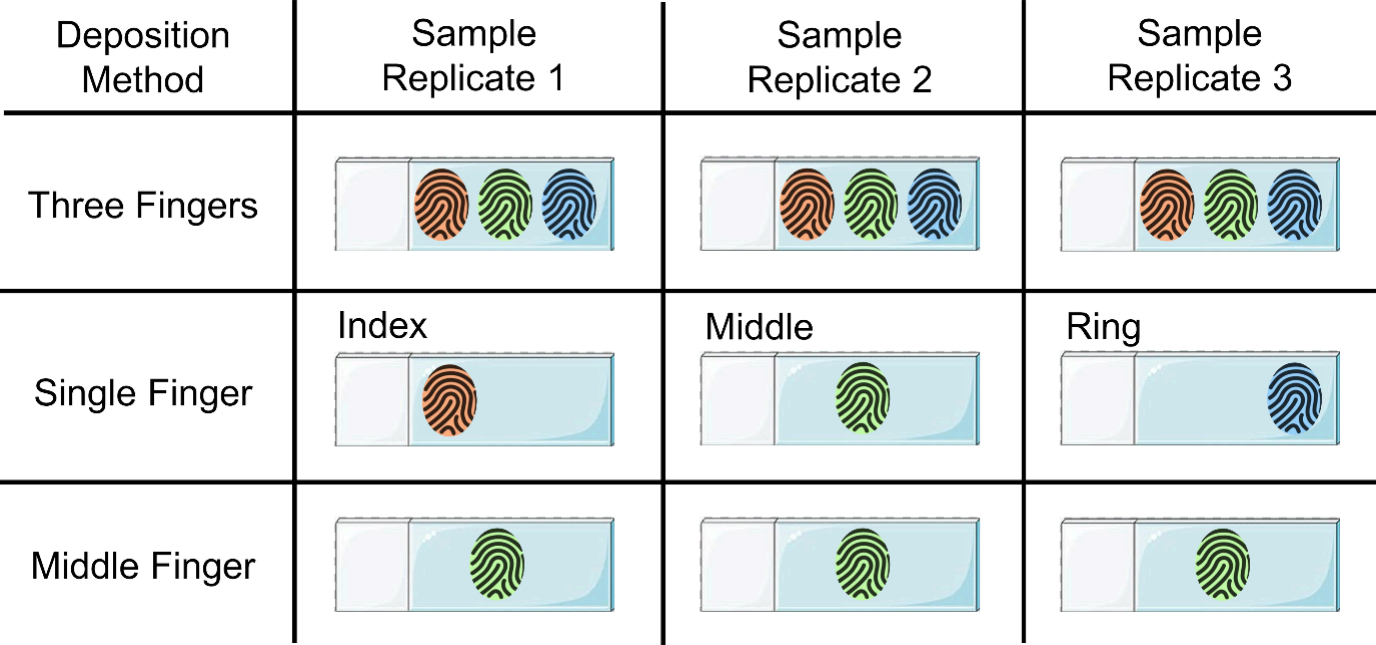
Three deposition methods for fingermark residue analysis. Three fingers consisted of the index, middle, and ring fingers of one hand. A singer finger deposition consisted of replicates of individual depositions from an index, middle, or ring finger. Middle finger depositions consisted of replicates of the same individual finger’s residue, a middle finger.

### GC×GC-TOFMS Conditions

For initial GC-TOFMS and GC×GC-TOFMS analyses, oven temperatures and conditions are listed in Table 1. GC-TOFMS samples were run with a mass spectral acquisition rate of 20 spectra/s and two-dimensional samples were run with a 200 spectra/s acquisition rate to reflect the difference in peak width from the two techniques. The mass range was set to 35–500 *m*/*z* with an extraction frequency of 32 kHz for both the 1D and 2D detections. The electron ionization energy used was 70.0 eV.

**Table 1:** Initial Parameters Used for GC-TOFMS and GC×GC-TOFMS Analysis of Fingermark Residue Using a Pegasus BT 4D with Dual-Stage Cryogenic Thermal Modulation^a^

GC parameters	GC-TOFMS	Pegasus BT 4D GC×GC-TOFMS
Column(s)	Rxi-5MS (30 m × 0.25 mm i.d. × 0.25 μm)	^1^D: Rxi-5MS (30 m × 0.25 mm i.d. × 0.25 μm)
		^2^D: Rxi-17Sil MS (0.9 m × 0.25 mm i.d. × 0.25 μm)
Modulation period	N/A	3 s (0.9 s hot pulse, 0.6 s cold pulse)
Oven temperature	40 °C (hold 1 min) to 250 °C at 5 °C/min (hold 2 min)	
Transfer line temperature	250 °C	
Secondary oven offset	N/A	+5 °C
Modulator	N/A	+15 °C
Carrier gas	Ultrahigh purity (UHP) helium at 1.00 mL/min	

a Note that the GC-TOFMS method was run with secondary column installed but modulator disabled so that 1D and 2D output would be comparable.

For method development, six programmable GC×GC parameters were compared with two to three options each (Table 2). This workflow was adapted from Fisher and Perrault Uptmor (2024) and is described further.^21^ Results for each comparison were investigated individually by comparing all options for each parameter based on resolution capacity and ability to resolve coelutions. Each metric was tested using the same sample that was acquired, as described above.

**Table 2:** Parameters for Optimization of a Nontargeted Analysis Method for Fingermark Residue on a Pegasus BT 4D GC×GC-TOFMS

parameter	option A	option B	option C
Modulation period	3 s	4 s	5 s
Hot pulse time	1 s	1.2 s	1.5 s
Hold time at oven start	1 min	2 min	5 min
Hold time at oven end	8 min	10 min	12 min
Oven ramp rate	5 °C/min	10 °C/min	15 °C/min
Secondary oven offset	+5 °C	+10 °C	N/A

### Data Processing

Samples were processed in ChromaTOF software version 5.56 with a data processor version 1.2.0.6 (LECO Corporation, Saint Joseph, MI). Peaks in 1D GC samples were identified with a minimum signal/noise ratio of 10. In all GC×GC samples, peaks were identified using a minimum signal-to-noise ratio of 700 and a minimum stick count of 3. Mass spectra were searched in the NIST Mass Spectral Library version 3.0, 2023, with a minimum spectral similarity of 700 for a match and relative abundance threshold of 10. ChromaTOF Tile (LECO Corporation) was used to determine analytes with the highest Fisher ratio (F-ratio) between donors in cosmetic application. Tile-based software allows for the class comparison of chromatograms to one another to extract relevant features that differentiate samples. Raw. SMP files were imported into ChromaTOF Tile and sorted based on *F*-ratio, a measure of class-to-class variation divided by within-class variation. High *F*-ratios represent analytes that are different between classes but consistent within a class. Samples were labeled according to the donor, resulting in five classes representing five donors with three replicates per class. A tile grid was overlaid in 2D chromatographic space to compare raw MS signals, which are considered “hits” if they meet the set thresholds. A tile size of 4 (^1^D) and 31 (^2^D) were autocalculated in the software based on average peak widths (^1^D) and heights (^2^D). A signal-to-noise threshold of 500 and *F*-ratio threshold of 20 were used to filter out low priority hits. Analyte identification was conducted using the mainlib library (NIST) and confirmed with ChromaTOF library matches. The hits were ranked in the hit list according to the highest *F*-ratio values. Hits were rejected and removed from further analysis based on the following criteria: (1) the hit was column bleed, (2) the same hit crossed multiple tile spaces, and (3) there were two or more hits within one tile so redundant hits were removed and only one hit would be accepted. The first 20 accepted hits were used in a principal component analysis (PCA) of the five donors using a built-in PCA function in the ChromaTOF Tile software.

## Results and Discussion

### Initial 1D and 2D Analyses

Initial samples were analyzed in 1D GC mode and then in GC×GC mode. Compound classes identified in 1D mode included fatty alcohols, fatty acids, wax esters, sterols, and precursors. In 1D mode, 15 compounds were identified using the NIST library (Figure 2A). Although some other peaks existed in the chromatogram, they either did not exceed the signal-to-noise (*S*/*N*) threshold of 10 or did not have a high enough library search value for confident identification.

**Figure 2:**
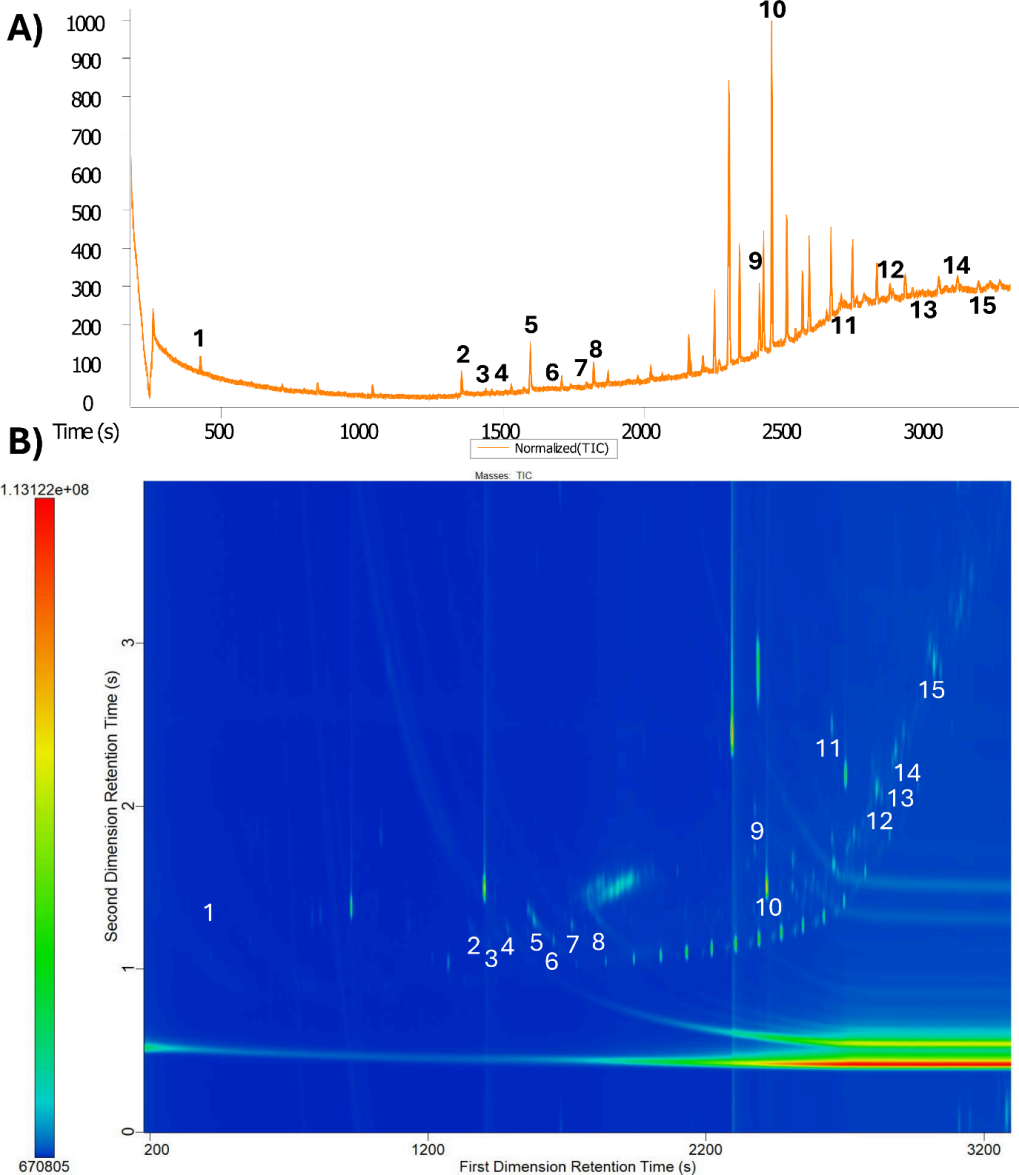
(A) Total ion current (TIC) of a one-dimensional chromatogram of a fingermark residue (normalized). Fingermark analytes of interest: (1) nonanal, (2) tetradecanoic acid, (3) isopropyl myristate, (4) pentadecanoic acid, (5) hexadecanoic acid, () isopropyl palmitate, (7) 1-octadecanol, (8) stearic acid, (9) 13-docosenamide, (10) squalene, (11) cholesterol, (12) palmitelaidic acid, (13) 9-tetradecenoic acid, hexadecyl ester, (14) 9-hexadecenoic acid, hexadecyl ester, (15) 9-hexadecenoic acid, octadecyl ester. (B) TIC contour plot of fingermark residue, same volunteer as (A), analyzed in GC×GC mode.

When analyzed in GC×GC mode, analysis resulted initially in 13 new analytes detected without any optimization of instrumental parameters. An initial primary oven temperature increase resulted in 15 additional detected analytes. A modulation period increase resulted in six additional long chain fatty acid esters that could not be distinguished by their mass spectra but were still chromatographically resolved. One major benefit of the two-dimensional separation provided by GC×GC is the ability to chromatographically resolve these components, which are highly similar in structure but have minimal differentiation in their mass spectra. Five additional cosmetic contaminants were resolved using GC×GC: avobenzone, octocrylene, 2-ethylhexyl salicylate (octisalate), diisopropyl adipate, and α-tocopheryl acetate, which are discussed in detail later in this study. The biological sterol cholesterol and its precursor, squalene, were identified after increasing the initial and final primary oven temperature. Squalene is a precursor molecule to the biologically important cholesterol; it is the highest reported lipid found in fingermark studies using GC-MS methods as well as other analytical techniques, so it was used as an analyte of focus throughout method development within this study.^16,22^ A previous study analyzing cosmetic compounds in fingerprints found coelutions between the cosmetics of interest and fingerprint compounds.^9^ This could be true for other forensic analytes of interest present in fingermarks such as condom lubricants, gunshot residue, illicit drugs, explosives, etc. The additional chromatographic space provided by the second dimension would allow for a nontargeted screening of forensic evidence with a higher confidence of resolving potential coelution of analytes (Figure 2B). The ability of GC×GC compared to 1D GC to resolve and identify additional exogenous compounds from fingermark residue lays the foundation for future forensic research relating to identification of forensically relevant contaminants in complex samples. In addition, the column bleed shown at the bottom of the contour plot is sufficiently separated from other analytes in the sample, increasing the signal-to-noise ratio of low level analytes that may be obscured by higher baseline later in the chromatographic run. In traditional GC-MS, there is no separation of chromatographic artifacts like column bleed from other analytes in the sample if they are found at the same retention time. The results displayed in Figure 2 were representative of all samples run by using equivalent methods.

### Method Optimization

Resolution and sensitivity were maximized for the widest range of analytes in this optimization. Method optimization of fingerprint residue for GC×GC analysis with cryogenic thermal modulation included six optimized parameters: modulation period, hot pulse time, primary oven hold time at the beginning of the run, primary oven hold time at the end of the run, oven ramp rate, and secondary oven offset. Instead of optimizing the modulator temperature offset, a recommended offset of +15 °C was consistently used.

Modulation period is one of the most important parameters to optimize for GC×GC methods. Hot pulse time (linked with cold pulse time) is a specific parameter only relevant for thermal modulators, such as the cryogenic dual-stage modulator used in this study. Modulation periods of 3, 4, and 5 s were tested using base method parameters described above. A method with modulation period of 3 s produced five peaks that exhibited wraparound (Figure 3A), indicating that the method did not have a long enough modulation period. Wraparound occurs when an analyte from one modulation period is still separating in the second dimension when the next modulation period is introduced to the ^2^D column.^23^ These wrapped around analytes can be split between the very bottom and the very top of the contour plot or displayed entirely in the bottom of the plot for the next modulation period. They do not reflect their true first dimension or second dimension retention times, making analyte identification by retention matching problematic. In addition, remaining in the secondary column for a second modulation period results in significant band broadening that will impact the *S*/*N* of the peak. A second increase from 4 to 5 s removed all wraparound peaks (Figure 3B,C). This longer modulation period is consistent with a study from Ladislavová et al. (2023) which also utilized a GC×GC system with a cryogenic thermal modulator at 6, 8, and 10 s to analyze fingermark residue found on fired gun cartridge cases.^19^ This longer modulation period improved upon the targeted method used by Kindell and Bridge (2023), which utilized a short modulation period of 0.9 s to target seven fingermark analytes.^20^ Using a longer modulation period for a nontargeted method will improve the ability to resolve fingermark analytes as well as any contaminant analytes in a forensic screening analysis. Hot pulse time of a chromatographic method relates to the time of firing for the two hot jets in the quadjet thermal modulator. Three hot pulse times were tested: 1.0 1.2, and 1.5 s hot pulse. Lengthening the hot pulse time also subsequently shortens the cold pulse time, since the sum of the cold and hot jet firing should equal one-half of the modulation period in a two-stage modulator. Peak shape of analytes using a 1.5 s hot pulse time were most circular (i.e., Gaussian in one dimension) (SI Figure 1), so 1.5 s was chosen resulting in a 1.0 s cold pulse time. A modulation period of 5 s and a hot pulse time of 1.5 s were chosen for further analysis.

**Figure 3:**
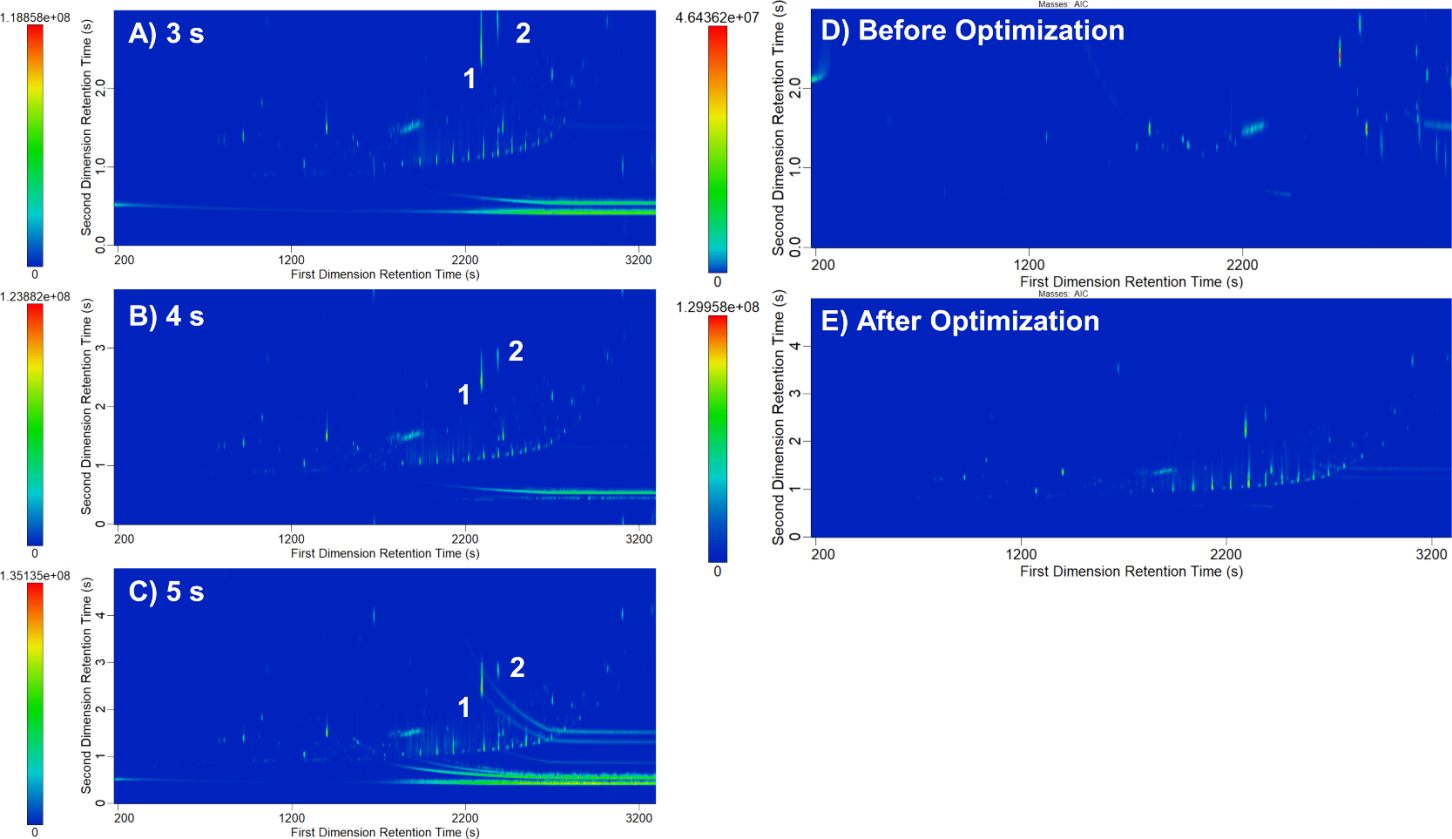
Overview of three total ion current (TIC) contour plots of methods using different options for the modulation period: (A) 3 s modulation; (B) 4 s modulation; (C) 5 s modulation. Peaks 1 and 2 exhibit wraparound in (A). Analytical ion current contour plots of fingermark residue (D) before optimization and (E) after optimization. Note: *x-* and *y*-axes are different due to different chromatographic parameters.

Chromatographic oven settings were optimized for three oven settings: initial hold time, final hold time, and oven ramp rate. A longer temperature hold was not necessary at the beginning of the run due to the absence of fingerprint analytes at low ^1^D retention times (SI Figure 2). Three hold times at the end of the run were evaluated. Based on initial GC-MS analysis, fingermark analytes eluted at higher retention times so therefore longer hold times of 8, 10, and 12 min were tested. A longer hold time of 10 min was chosen for complete elution of fingermark analytes from the column (SI Figure 3), similar to a long hold time of 7 min from the only other published study of fingermarks using GC×GC.^20^ Three oven ramp rates were compared for optimization of the fingermark residue: 5 °C/min, 10 °C/min, and 15 °C/min. Total ion current (TIC) contour plots of faster ramp rates (10 °C/min and 15 °C/min) exhibited high analyte peak tailing (SI Figure 4B,C). A 15 °C/min ramp rate displayed a loss of resolution for analytes compared to the 5 °C/min rate (SI Figure 4C). Although some wraparound was present within a 5 °C/min ramp rate and absent in 10 °C/min and 15 °C/min options, other chromatographic parameters of this tested method including modulation period were carried out with the established base method and not the optimized version. The optimized modulation period of 5 s was longer than the base modulation period of 4 s, which would be expected to alleviate this wraparound. A ramp rate of 5 °C/min was chosen.

The last parameter optimized, the secondary oven offset, was specific to GC×GC systems with a secondary oven that houses the ^2^D column. The temperature of the secondary oven and ^2^D column should be higher than that of the primary oven and ^1^D column due to the use of cold and hot jets to facilitate carrier gas/analyte flow compression and reinjection onto the ^2^D column. An offset temperature of +10 °C was chosen for the optimized method due to the absence of wrapped around peaks (SI Figure 5). Higher temperature options for the secondary oven offset were consistent with longer modulation and hot pulse times as well as a longer hold time at the end. The higher temperatures and longer hold times are consistent with other GC×GC studies that analyzed fingermark residue which ramped to a final temperature of 320 °C with 7 min end hold^20^ and 10 min end hold.^19^ The chosen optimized method included the following parameters: 5 s modulation period, 1.5 s hot pulse time, 1 min hold time at the beginning of run, 10 min hold time at the end of run, 5 °C/min oven ramp rate, and a +10 °C secondary oven offset. A fingermark residue contour plot before optimization (Figure 3D) shows wraparound and incomplete elution of compounds from the column before the run time is over compared to a contour plot displaying fingermark residue analyzed with the optimized method (Figure 3E).

### Sample Extraction Optimization

Two methods for extraction of the fingermark off the microscope slide were compared, using reproducibility and number of extracted analytes as metrics for evaluation. Percent relative standard deviation (RSD) for the pipet agitation method were consistently higher than those for the cotton swab extraction (Table 3). The lower % RSD of the cotton swab extraction indicated less variance and, consequently, a more reproducible extraction method. The cotton swab method also increased the number of analytes detected from 35 to 70 compounds (Figure 4A). Steroids/sterol and pentacyclic terpene classes totaled around 33% of the new total number of compounds detected after the cotton swab method. These classes exhibited heavier molecular weights and were less volatile, which would hinder their extraction in the pipet agitation extraction method; hence, they were not detected using that extraction method. The cotton swab method was used for future extraction of fingermark residue due to more accurate reproducibility measured by lower % RSD values and a higher number of analytes/compound classes detected.

**Table 3:** Percent Relative Standard Deviations of Two Sample Extraction Methods, Pipette Agitation (*n* = 6) and Cotton Swab Extraction (*n* = 8), for Six Fingermark Analytes

analyte	pipette agitation	cotton swab extraction
squalene	17	9
cholesterol	25	15
isopropyl palmitate	28	19
1-octadecanol	40	25
octocrylene	38	34
octisalate	23	22

**Figure 4:**
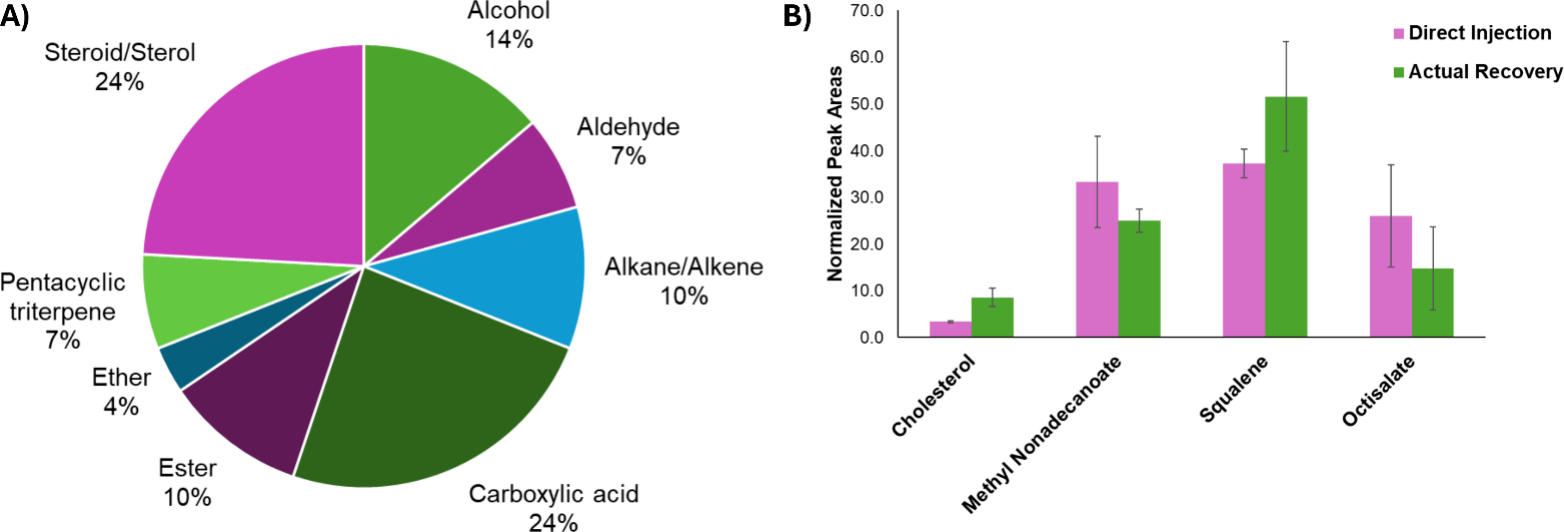
(A) Pie chart displaying compound classifications of 29 additional fingermark residue compound classes found after cotton swab extraction compared to pipet agitation extraction method. (B) Recovered amount of four fingermark standards from a 500 ppm spiked microscope slide extraction, reported as normalized to the total peak area of the four analytes (*n* = 3). Direct injections of 1 μL of liquid injections of the same four standards, normalized to the total peak area of the four analytes.

A recovery study was conducted to evaluate the effectiveness of the determined extraction method at transferring the residue from the microscope slide to the liquid injection form. Four standards at 500 ppm, including two sterols, one fatty acid methyl ester, and one exogenous compound, were spiked on a microscope slide. The standards underwent the same extraction process outlined above in the methods. The amount of standard recovered after the extraction procedure was normalized to the total peak area of the four standards. A comparison was made between the 1 μL direct injection of 500 ppm standard into the instrument and 1 μL of the same concentration spiked onto a microscope slide (Figure 4B), using peak area comparisons to represent recovery. Error bars represent the variability from replicate extractions. Analytical variability, including instrument response, could also account for additional variability within the recovery values. Recovery values for the four analytes were within reasonable recovery ranges (Figure 4B).

Fingermarks from three fingers were analyzed in a proof-of-concept study design to confirm adequate resolution and sensitivity for the method development and optimization described above. In forensic applications of fingermark residue analysis, the forensic evidence quantity may be highly variable. To address a more realistic evidence scenario, a single fingerprint deposition study was investigated to determine whether differences in the detection capability were present using this analysis method. Depositions from three fingers were compared to replicate depositions from a single but variable finger (including pointer, middle, and ring single finger deposition of residue) and replicate depositions from just middle finger residue. Five fingermark analytes were used for comparison across deposition types: squalene, 1-octadecanol, cholesterol, isopropyl myristate, and isopropyl palmitate. Across three of five analytes, the three finger deposition method exhibited the lowest % RSD values (Table 4). Consistent with other studies, squalene was the highest abundant analyte across all methods.^4,24^ Squalene has been used as the reference for internal normalization to an analyte because of its consistency as a large peak or the largest peak in a fingermark chromatogram using GC-MS analysis.^4,13,16^ In this study, squalene exhibited the lowest % RSD values for two of the three deposition methods, while 1-octadecanol, the second highest abundant analyte investigated in this study, exhibited the most consistent % RSD values across the three deposition methods. Cholesterol, isopropyl myristate, and isopropyl palmitate % RSD values were higher in comparison and demonstrated greater variability (Table 4). The much lower abundances of the latter three analytes could contribute to greater variability in the detection and peak area calculation. Cholesterol is a lower abundance analyte and has been shown to be present in lower levels in adult subjects compared to children.^3,8^ Since this study was performed on individuals over the age of 18, the lower abundances of cholesterol are consistent with the age of the study subjects. Squalene and cholesterol are excreted from sebaceous glands in the skin where squalene is less readily converted to cholesterol than within other skin gland types (eccrine or apocrine).^6,10^ This lack of conversion of squalene into cholesterol could explain consistently low relative abundances of cholesterol compared to consistently high relative abundances of squalene. High variability could be minimized by additional studies with a greater number of replicates. A limit of detection (LOD) study in the future could serve as a solution to determine whether these low-level concentrations can be accurately detected in a nontargeted screening approach to fingermark residue evidence.

**Table 4:** Percent Relative Standard Deviations for Squalene, 1-Octadecanol, Cholesterol, Isopropyl Myristate, and Isopropyl Palmitate Deposited with Three Fingers (*n* = 8), a Single Finger (*n* = 6), or a Middle Finger (*n* = 10)

deposition method	squalene	1-octadecanol	cholesterol	isopropyl myristate	isopropyl palmitate
Three fingers	7.09	26.88	13.70	66.96	10.47
Single finger	19.38	21.53	17.20	18.94	42.00
Middle finger	18.71	23.52	35.64	25.15	30.49

To determine whether there were significant differences between peak areas from three fingers compared to a single-finger or middle finger deposition, an *F*-test and *t* test were used between three finger and single-finger groups, three finger and middle finger groups, and single and middle finger groups. Peak areas were statistically different between three-finger deposition and single-finger deposition for four of five analytes: squalene, 1-octadecanol, cholesterol, and isopropyl palmitate (*p* < 0.001 for all four analytes) (Figure 5A,B). Isopropyl myristate peak areas did not exhibit statistical differences, likely due to the large variability of extracted peak areas. Variability within this study was derived from the variability of each fingermark deposition and the analytical variability from analyzing a set of samples over time. Biological variability within a set of donors includes interdonor variability of samples from different donors and intrasample variability between samples from the same donor. Both intra- and intervariability have been reported as relatively high in the literature in human subjects.^10,12,13,25^ A single finger would be likely one-third of the residue volume of a three-finger deposition, leading to a statistical difference in peak areas. Peak areas were statistically significant between three fingers and middle finger residue for the same four analytes: squalene, 1-octadecanol, cholesterol, and isopropyl palmitate (*p* < 0.001) (Figure 5A,B). Although average peak areas were larger for three finger deposition compared to the middle finger deposition method, large variability (standard deviation) in the three finger deposition peak area for isopropyl myristate could be a contributing factor for the analyte without significant difference. One way to minimize variability from different donors or depositions would be to report a ratio of two analytes of interest instead of relative or absolute quantitation.^6,13^ Another way to reduce the analytical variability is to introduce an internal standard that would serve as the compound used to calculate relative peak areas. Internal standards such as decane or 2-octanol^26^ and 1-decanol^10^ have been used to study targeted fingermark classes using GC-MS. However, these internal standards are lighter than most fingermark analytes (i.e., do not elute at the same retention times as most fingermark analytes in a nontargeted study) and therefore would be less appropriate when studying quantitation of heavier analytes. Other known fingermark analytes such as methyl nonadecanoate have been used as an internal standard when the goal of a study includes absolute quantitation of a subset of fingermark analytes classes that exclude the chosen internal standard.^6^ Neither of these two scenarios pertains to this study, so an internal standard was not used. A heavier internal standard such as anthracene^13^ could be beneficial for future studies, which could serve as a reference peak for normalization to minimize variability contributed by the analytical technique. Due to the nature of the sampling method, this would need to be added at the reconstitution step in the procedure.

**Figure 5:**
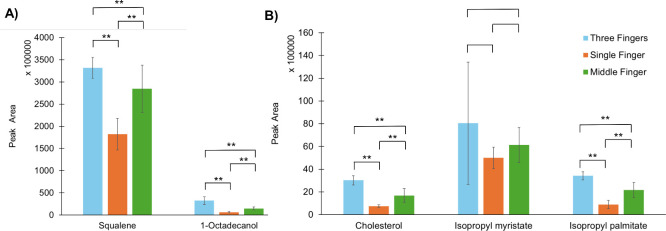
Peak areas of (A) squalene and 1-octadecanol; (B) cholesterol, isopropyl myristate, and isopropyl palmitate from three finger, single-finger, and middle finger depositions. Error bars represent standard deviations. Statistical significance between deposition methods denoted by asterisked brackets. * denotes *p* < 0.05. ** denotes *p* < 0.001. Note: *y*-axis scales are different between (A) and (B).

Single finger depositions exhibited lower average peak areas than middle finger depositions. Squalene, 1-octadecanol, cholesterol, and isopropyl palmitate peak areas were statistically significant between single finger and middle finger deposition (*p* < 0.001 for the four analytes) (Figure 5A,B). Single finger variation included variability within the three fingers used for deposition; single finger depositions were performed using the index, middle, and ring fingers of each hand. Although each residue consisted of one fingermark, replication was performed using three fingers which could be an additional source of variability as cited by other fingermark studies.^12,13^ The middle finger depositions, consisting of one repeated finger deposit, were aimed to investigate this variation in finger type. A study comparing replicates of residue from each of the five fingers would be useful for determining variation between fingers.

### Exogenous Compound Screening

The optimized method for analyzing fingerprints was applied to a set of fingermarks from five donors to screen for exogenous component contamination and variability between donors. Replicate samples from each of the donors displayed a relatively low intrasample variability for the common fingerprint residue analytes of squalene and cholesterol. Donor 4 displayed the lowest intrasample variability of the five subjects with % RSD values for cholesterol and squalene of 9.10% and 8.40% respectively. The highest intrasample variability was displayed in donor 2 with 38.18% and 22.70% for cholesterol and squalene. Cholesterol was detected in one of three replicate residues from donor 3, and squalene was detected in two of three replicate samples from that donor; therefore, sample size was not large enough to calculate % RSD. Sweating characteristics, the donor themselves, deposition force, and the portion of the finger touching the substrate where residue was deposited are known to be factors that categorize a fingermark donor into strong, medium, or weak donation abilities.^25,27,28^ In this study, volunteer 3 could be designated as a “weak” fingerprint donor, having a decreased ability to deposit residue onto a surface or an uneven deposition force when depositing fingermarks. Intradonor variability measured by % RSD values was consistently lower (i.e., less variable) than interdonor variability in the literature^10,12^ and also within this study. A larger study targeting a wider range of analytes would support this finding.

Residues from three donors contained exogenous compounds with a relatively high variability. Five exogenous analytes were resolved from fingerprint residue: diisopropyl adipate (cosmetic), octisalate (sunscreen), octocrylene (sunscreen), avobenzone (sunscreen), and α-tocopheryl acetate (vitamin E acetate) (Figure 6C). α-Tocopheryl acetate can be found in moisturizing cosmetic products such as vitamin E acetate. The presence of vitamin E acetate could indicate that these three donors had contact with moisturizing cosmetic products on the day of sampling. Avobenzone, octocrylene, and octisalate were found in five residues originating from one donor. These three analytes are UVB blockers commonly found in sunscreen ingredients.^17^ The presence of three UVB blockers in one donor and the absence of those analytes in all other donors in this study could be used to infer lifestyle choices of sunscreen wear (Figure 6A). A previous study found that UVB blockers avobenzone, octocrylene, oxybenzone, and octinoxate were markers for differentiation between sunscreen brands.^14^ Therefore, exogenous compounds not only could be used to indicate lifestyle preferences of a criminal suspect but could link evidence to a suspect based on brand identification. The presence of the three UVB blockers avobenzone, octocrylene, and octisalate indicated that this donor most likely wore or came into contact with sunscreen on the day(s) of residue sampling or close to then. Additional study on the persistence of these analytes in washed hands would be valuable to make additional conclusions. The high variability within samples from the same donor makes it challenging to set absolute quantitative cutoffs for forensic casework. The ability to resolve those compounds of interest in fingermark residue with ample chromatographic space for resolution of potential anthropogenic analytes of interest is a benefit of using a nontargeted analysis for a screening of forensic evidence, even with high variability between individual donors. The identification and presence of trace analytes that could link a suspect to a crime scene or crime are an important component of trace evidence analysis.

**Figure 6:**
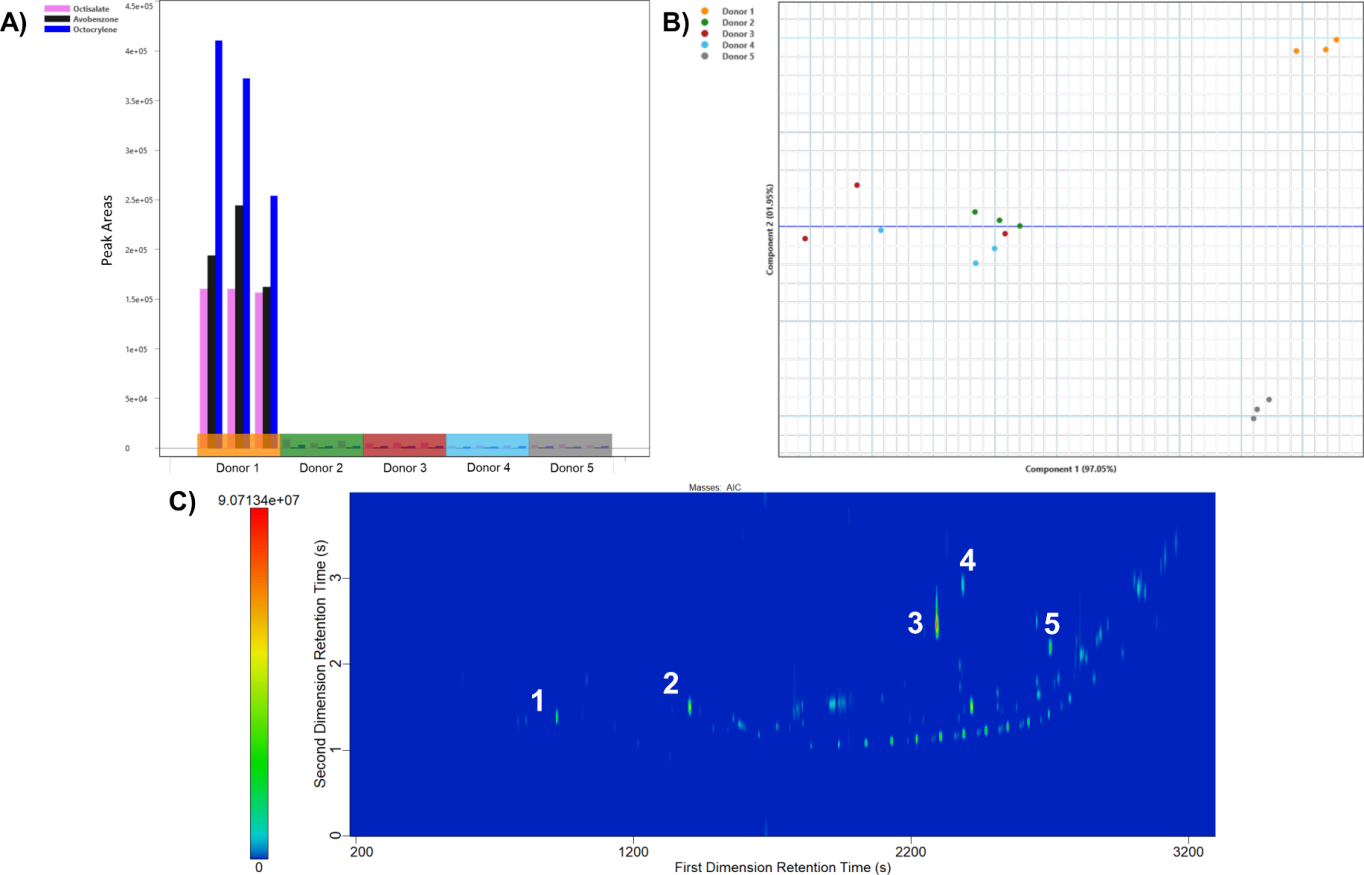
(A) Presence of UVB blockers found in three replicates of one fingermark donor. Replicate samples are grouped by colored blocks on the *x*-axis. (B) Principal component analysis (PCA) of five fingermark donors (*n* = 3). (C) Analytical ion current (AIC) of donor with cosmetic contamination. Cosmetic peaks: (1) diisopropyl adipate, (2) octisalate, (3) octocrylene, (4) avobenzone, and (5) α-tocopheryl acetate.

Variability was high for these three UVB blocking analytes despite the fact that they originated from the same donor. Avobenzone demonstrated the highest variability (73.24% RSD) compared to octisalate (23.20% RSD) with the lowest. An investigation of active ingredients of bug sprays and sunscreens also found inconsistent variability across six studied sunscreen ingredients.^14^ Donors 1 and 2 deposited more reproducible fingermarks, as seen by the clustering of replicates in Figure 6B by PCA of 20 analytes with the largest *F*-ratio between donors. This differentiation of cosmetic products between donors could be useful to forensic investigators in the future and demonstrate the success of a nontargeted screening for analyzing fingermarks. It should be noted that other studies in the literature have investigated cosmetic contamination of fingermarks as a secondary study goal; two studies with the goal of chemical profiling of fingermarks used cosmetic presence in residue as a case study of fingermark contamination.^5,16^ This type of method would also be extremely beneficial in the case of cosmetic products that contain terpenes and terpenoids, such as some allergens found in fragrance products. In those cases, some components are challenging to differentiate based on their mass spectra due to their similarities in 70 eV electron ionization mass spectra. Being able to chromatographically resolve such components from one another and differentiate them based on retention time or retention index would provide an added benefit of using a higher resolution separation in advance of TOFMS detection. The benefit of using a nontargeted screening method for forensic evidence analysis could be the ability to detect and identify analytes outside of a targeted few that may still be relevant or influential in criminal investigations. The use of such a nontargeted method as a screening tool in forensic laboratories could enhance current workflows or additionally eliminate the need for multiple analyses of a single evidence sample.

## Conclusion

An initial comparison between 1D GC and GC×GC found GC×GC to be beneficial for fingermark analysis due to higher sensitivity and increased peak capacity for potential forensic investigations. A nontargeted method for GC×GC analysis was modified from the literature sources and optimized experimentally. Sample extraction of the fingermark samples from collection on microscope slides was evaluated for reproducibility. For consideration of more realistic forensic evidence, three finger sample depositions were compared with single finger depositions. Single finger depositions were found to be significantly lower in extracted peak areas but still produced a detectable signal. A LOD study based on fingermark analyte standards should be the focus of future work to determine qualitative threshold values that would be useful for forensic investigations.

Exogenous compound screenings could be useful in forensic investigations. Personal care products such as those found in this study could aid in lifestyle assessments of criminal suspects. In a nontargeted assessment of collected fingermark samples in this study, three sunscreen compounds and two moisturizer compounds were found in a study of six subjects; these were detected after the subjects had washed their hands and touched their face prior to deposition. Only one subject’s fingermarks contained UVB blockers found in sunscreens, and fingermarks from three subjects contained components commonly used in cosmetic moisturizers. These personal care product differences between individuals could be differentiated in fingermark samples using PCA to group subjects. Nontargeted screening of fingermark residues with an increased peak capacity has vast forensic implications. The extra chromatographic space provided by GC×GC combined with a nontargeted method optimized for fingermark analytes could provide a screening tool for routine forensic laboratories for lifestyle choices such as cosmetics but also for discovery of other forensically relevant compounds such as gunshot residue or illicit drugs that are present in fingermarks from touch chemistry. Further research on the analytical robustness and reliability of this method to analyze larger data sets with more donors and/or replicates would improve this nontargeted method.

## Supplementary Material



## Data Availability

The data underlying this study are not publicly available due to the procedures outlined in the PHSC approval. The data are available from the corresponding author upon reasonable request.

## Notes

Contour plots for five experimentally optimized instrument parameters for comprehensive two-dimensional gas chromatography analysis of fingermark residue (PDF)
