# Supplementary material for: Nontargeted Screening of Fingermark Residue Using Comprehensive Two-Dimensional Gas Chromatography–Time-of-Flight Mass Spectrometry for Future Use in Forensic Applications
Source: J Am Soc Mass Spectrom. 2025 Sep 9;36(10):2299–309. doi: 10.1021/jasms.5c00258 (PMC12492389; doi:10.1021/jasms.5c00258)
Supplement: Supplementary file 1 [file js5c00258_si_001.pdf]

Supplementary Information

Nontargeted screening of fingermark residue using comprehensive two-dimensional gas chromatography – time-of-flight mass spectrometry for future use in forensic applications

Emma L. Macturk[1], Katelynn A. Perrault Uptmor[1*]
[1]*Nontargeted Separations Laboratory, Chemistry Department, William & Mary*

*Corresponding Author
Katelynn A. Perrault Uptmor
Nontargeted Separations Laboratory, Chemistry Department
William & Mary
Integrated Science Center 1053
540 Landrum Drive
Williamsburg, VA 23188
kaperrault@wm.edu
757-221-4793

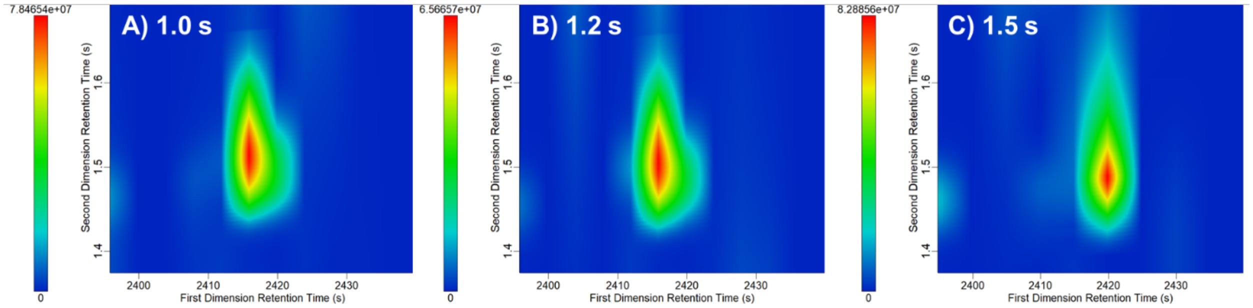

SI Figure 1. Analytical ion current (AIC) contour plots of three peaks representing squalene analyzed using three chromatographic methods with different hot pulse times: A) 1.0 s hot pulse, B) 1.2 s hot pulse, C) 1.5 s hot pulse.

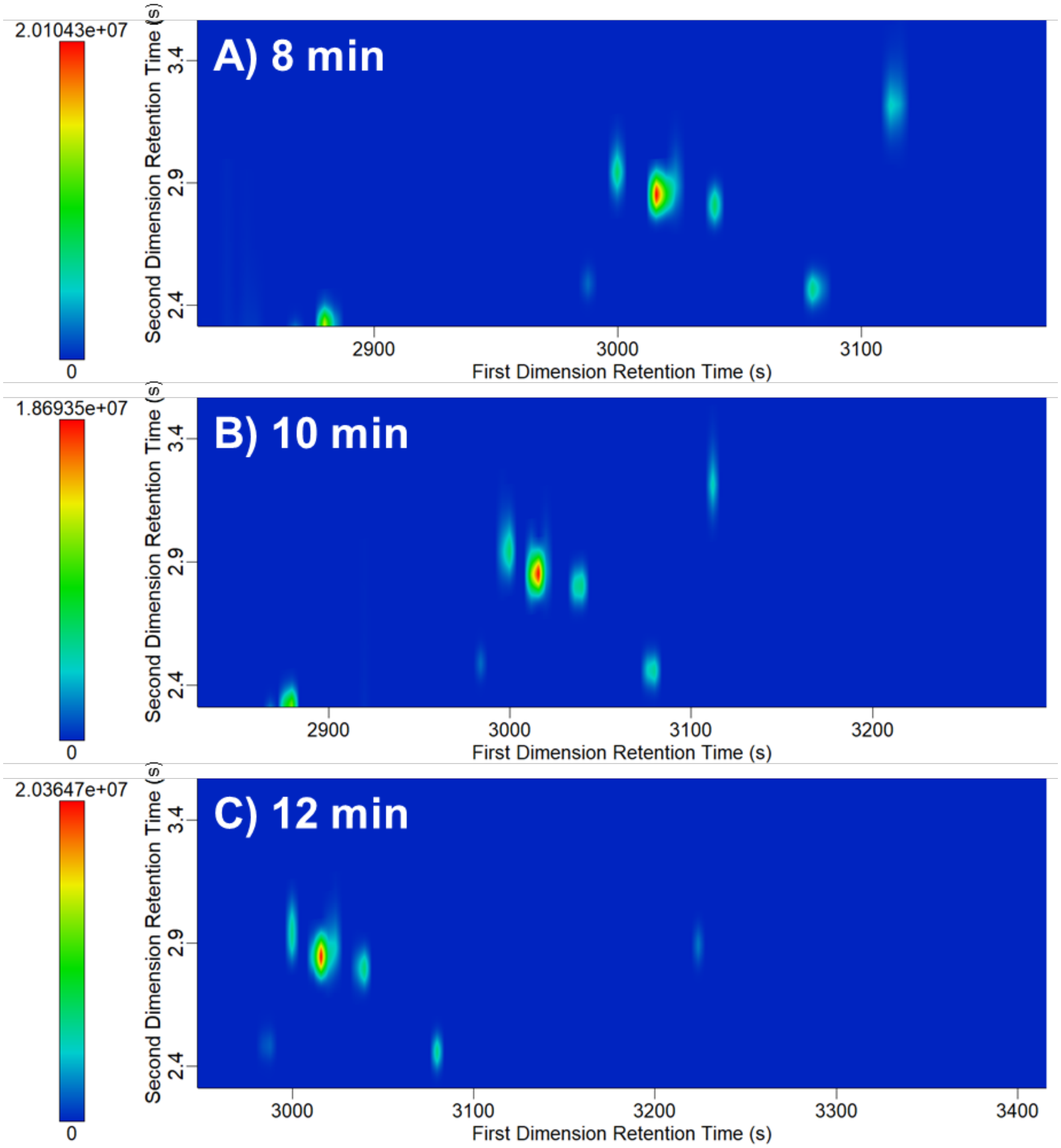

SI Figure 2. Analytical ion current (AIC) contour plots of three chromatographic methods using three initial temperature hold times: A) 1 min hold, B) 2 min hold, C) 5 min hold.

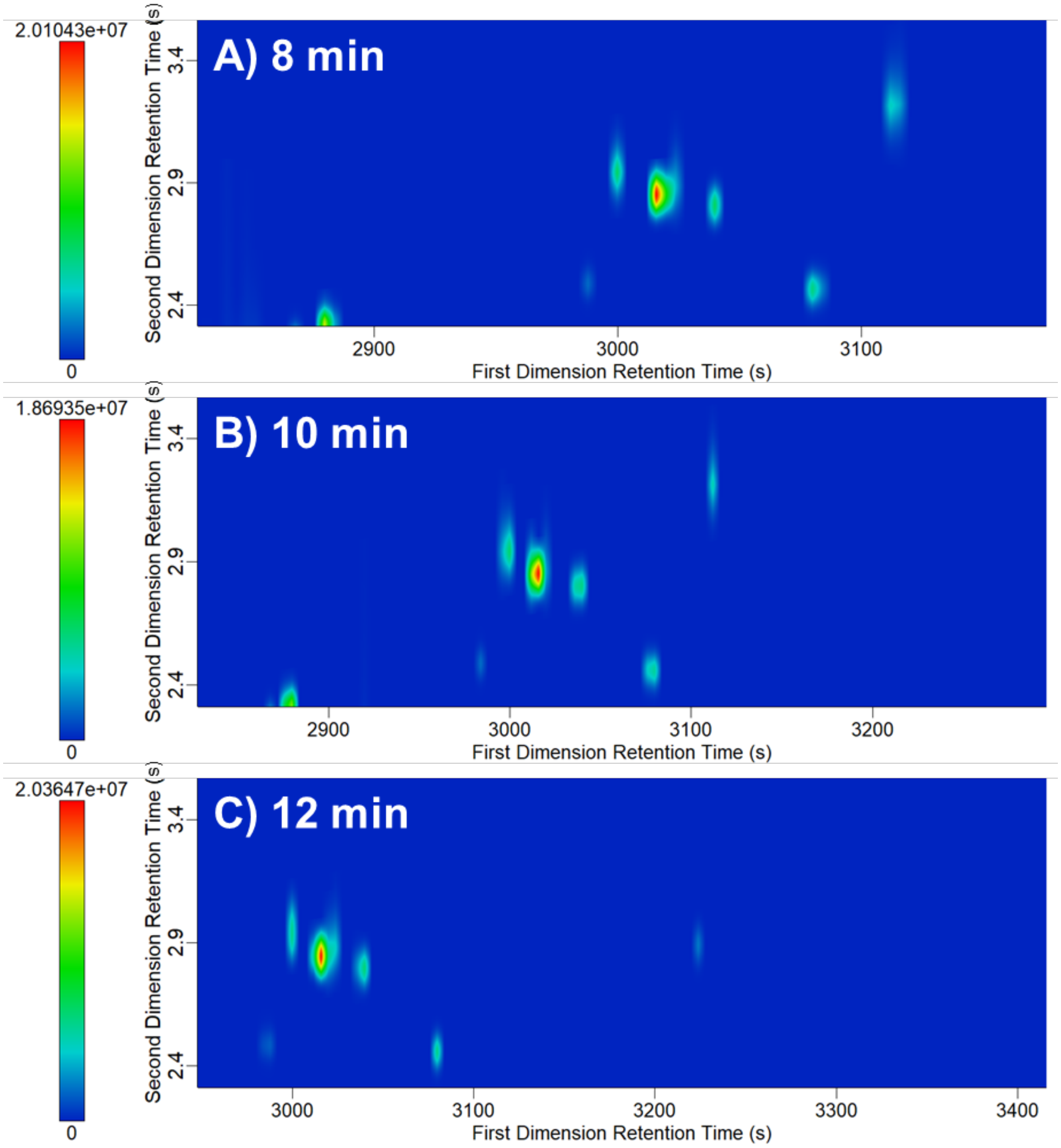

SI Figure 3. Analytical ion current (AIC) contour plots of three chromatographic methods using three final temperature hold times zoomed into the highest retention times: A) 8 min hold, B) 10 min hold, C) 12 min hold. Note the *x*-axes are different due to different run times.

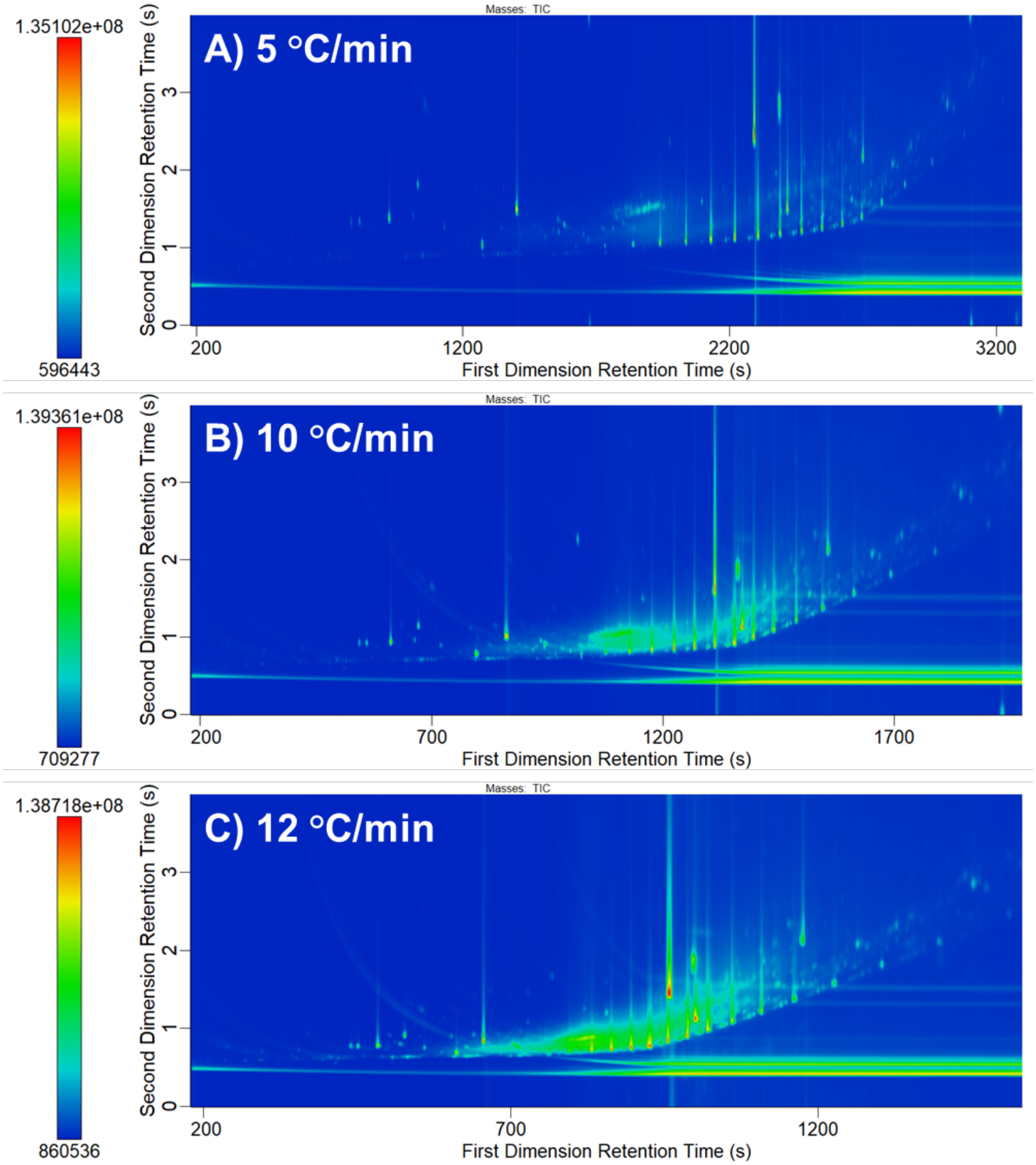

SI Figure 4. Overview of three total ion current (TIC) contour plots of methods using different options for oven ramp rate: A) 5 °C/min, B) 10 °C/min, C) 15 °C/min. Note: the *x*-axes are different due to different run times.

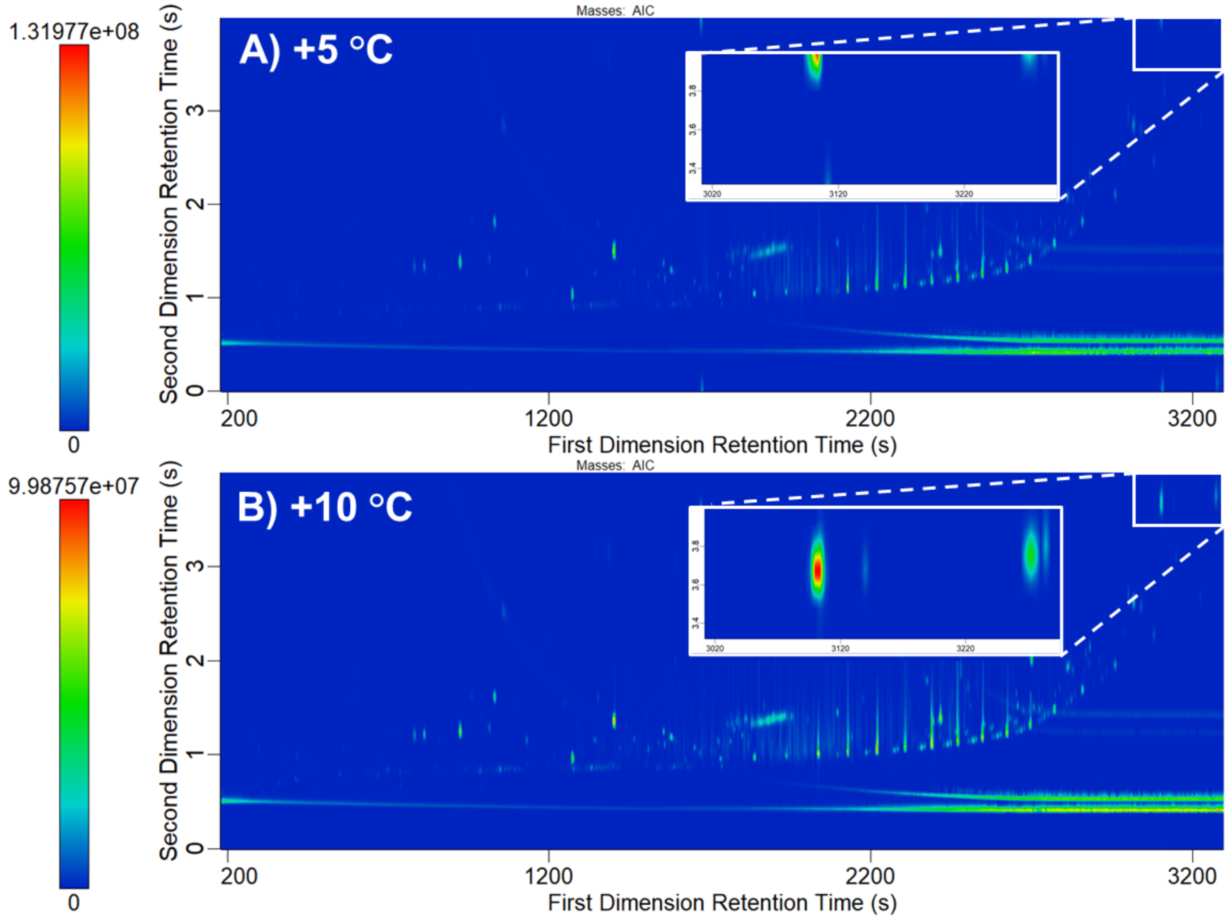

SI Figure 5. Comparison of analytical ion current (AIC) contour plots with two secondary oven offset temperatures: A) +5 °C, B) + 10 °C. Inlaid chromatograms display wrap-around peaks in A) and peaks without wrap-around in B).
